# Understanding the health-related quality of life impacts of caring for children and adolescents with rare progressive life-limiting conditions: key challenges and future research priorities

**DOI:** 10.1007/s11136-025-03937-8

**Published:** 2025-03-04

**Authors:** Philip A. Powell, Jill Carlton, Tessa Peasgood, Fleur Chandler, Josie Godfrey, Emily Reuben

**Affiliations:** 1 Sheffield Centre for Health and Related Research, University of Sheffield, Sheffield, UK; 2 Duchenne UK, London, UK; 3 JG Zebra Consulting, London, UK

**Keywords:** Carers, Children, Rare disease, Health-related quality of life, Health technology assessment

## Abstract

The inclusion of health-related quality of life (HRQoL) impacts on informal carers in health technology assessments (HTAs) is lacking due, primarily, to a deficiency in evidence and methodological issues on how informal carer HRQoL is captured and incorporated into economic models. These issues are magnified in areas of significant burden, such as caring for children and adolescents with rare, progressive, life-limiting conditions. In this commentary we outline key challenges in measuring, and incorporating in HTA submissions, informal carer HRQoL data in rare, progressive, paediatric, life-limiting conditions and identify future research priorities in this space. We argue that: (i) a generic model of carer HRQoL is likely inadequate to characterise the HRQoL impacts in this population; (ii) the underlying evidence-base is underdeveloped, including understanding commonalities across conditions, impact beyond the primary carer, and differences over time; and (iii) methodological challenges in modelling informal carer HRQoL in cost-effectiveness analysis are inhibiting progress. A research agenda is proposed that addresses these challenges by focusing first on in-depth qualitative research to develop an appropriate, content valid ‘disease-group-specific’ model for understanding informal carer HRQoL in rare, progressive, paediatric, life-limiting conditions. This model can be used to inform the appropriate measurement of carer HRQoL in this population, which, alongside methodological research on addressing modelling challenges, can help to facilitate the recommended inclusion of informal carer HRQoL data in HTA submissions for children and adolescents living with rare, progressive, life-limiting conditions.

## Introduction

Health-related quality of life (HRQoL) can be understood as ‘a multidimensional concept that includes the physical, psychological and social functioning [or impacts] associated with an illness or its treatment’ [1]. Many chronic health conditions have HRQoL impacts not only on the person living with the condition, but on the people around them, including informal carers [2]. In the case of rare, progressive, paediatric, life-limiting conditions (e.g., Batten disease; Duchenne muscular dystrophy [DMD]; Sanfilippo disease) these informal carers are typically family members, such as parents. Impacts on the HRQoL of informal carers can be profound, change over time, and encompass a range of psychological, social, and physical factors, such as increased anxiety and depression, relationship breakdowns, and enhanced physical burden, amongst others [3, 4].

Appropriately assessing and recognising HRQoL impacts on informal carers is important, not just for the carers themselves, but also for treatment funding decisions [5]. Including HRQoL impacts on informal carers is encouraged by many health technology assessment (HTA) agencies, including the UK National Institute for Health and Care Excellence (NICE) who promote the inclusion of ‘all health effects, whether for patients or, when relevant, carers’ in their manual for health technology evaluations [6]. The inclusion of quantified informal carer HRQoL impacts in submissions to NICE is increasing, but still represents a minority of all submissions [7]. Manifold issues remain about if and how informal carer HRQoL should be modelled in submissions, including: whether sufficient high-quality evidence on carer HRQoL (including utility data used in economic models [8]) is available; how carer HRQoL is modelled relative to patient HRQoL; and whether a sufficient empirically evidenced case is made to justify the inclusion of HRQoL impacts on informal carer(s) for a particular health condition [9]. Such issues are likely to be magnified in rare, progressive, paediatric, life-limiting conditions, which may represent a ‘special case’ of carer burden and HRQoL impact, over and above general caring impacts for more common health conditions [10].

Moving towards a position of appropriately recognising and including the HRQoL impacts of rare, progressive, paediatric, life-limiting conditions on informal carers in HTA raises significant questions and highlights potential gaps in knowledge and capacity in this area [5]. We argue that these knowledge gaps should be systematically addressed by researchers in the HRQoL community to best support the inclusion and recognition of the significant impacts that caring for children and adolescents with rare, progressive, life-limiting conditions can have on informal carers. Outstanding questions that have not yet been adequately addressed, include, for example, whether there are aspects of HRQoL uniquely affected by being an informal carer of someone living with a rare, progressive, paediatric, life-limiting condition, compared to caring for someone with a common health condition? What evidence is needed to best capture, represent, and facilitate the inclusion of informal carer HRQoL in HTA submissions in the context of rare, progressive, paediatric, life-limiting conditions? How can informal carer HRQoL data be best modelled in submissions to HTA agencies in the context of rare, progressive, paediatric, life-limiting conditions?

In this commentary we outline several key challenges in measuring, and incorporating in HTA submissions, the HRQoL impacts of caring for children and adolescents living with rare, progressive, life-limiting conditions. We then identify future research priorities in this space. This is intended to reflect the state of current knowledge and opinion in the area and act as a springboard for future work. The authors of this commentary include those with lived experience caring for sons living with the rare, progressive, life-limiting condition DMD.

## Key challenges

There are at least three key challenges to appropriately understanding and measuring HRQoL in informal carers of children and adolescents with rare, progressive, life-limiting conditions in the context of HTA. We discuss each below. In the section that follows on future research priorities we indicate how HRQoL researchers may begin to address these challenges.

### Challenge #1: a generic model of HRQoL is likely inadequate for informal carers of children and adolescents with rare, progressive, life-limiting conditions

Generic models of HRQoL are designed to be applicable to as wide a range of people as possible. In this way, they allow for comparability using the same metric; a characteristic which is favoured by HTA agencies, such as NICE [6]. However, their broad applicability means that they are not always suitable for health conditions that are located at the extremes of either the extent of the HRQoL impacts observed or the rarity of the types of impacts experienced [11]. For informal carers, many models of HRQoL and/or associated measurement tools have been developed predominantly based on the idea of younger individuals caring for older relatives living with common health conditions, such as dementia [12]. Although there are exceptions, such as the Family Reported Outcome Measure (FROM-16) [13]. This comment extends to commonly-used generic preference-weighted measures (PWMs) developed specifically for informal carers, which are used to generate utilities for cost-effectiveness evaluations of treatments in HTA [14]. While some PWMs, such as the CarerQol-7D, incorporated informal carers of children in their development phase, these were a minority of carers only, and the health conditions were not specified [15]. Other commonly-used generic PWMs, such as the EQ-5D-3L or -5L, have been used with carers to support HTA submissions [7], without adequate content validity first being assured in these populations. It is thus possible that a more specific model of HRQoL may be required to characterise fully the impacts experienced by informal carers of children and adolescents living with rare, progressive, life-limiting conditions, and– due to the extremes of the HRQoL impacts observed– a generic approach (such as that of the EQ-5D) may not be suitable for this population.

### Challenge #2: existing evidence of the HRQoL impacts on informal carers of children and adolescents with rare, progressive, life-limiting conditions is limited

A second challenge facing the generation and inclusion of HRQoL impacts on informal carers in rare, progressive, paediatric, life-limiting conditions in HTA is the gaps in the evidence-base. While there are multiple studies estimating the impact of informal caregiving for children and adolescents living with rare, progressive, life-limiting conditions (e.g., [4, 16–19], the available evidence is limited in several ways. First, most studies tend to focus on specific health conditions in isolation, such as DMD, spinal muscular atrophy (SMA), or Pompe disease. The focus on specific health conditions (and their idiosyncrasies) is potentially problematic for HTA as it limits comparability, which is needed to evaluate treatment effects and impacts on HRQoL on a common scale [11]. Second, studies have typically assessed carer burden (or similar constructs) using generic informal carer measures, such as the Zarit Burden Interview [20], which were not necessarily developed for use in this particular population and whose content validity has not been assured [2]. Third, studies focus predominantly on primary informal carers only (normally mothers) and do not investigate the impact on wider family members, who may also be involved in informal care. This is a problem for HTA submissions, where the number of informal carers to include in an economic model (i.e., that are affected by the health condition) is difficult to define and often arbitrarily decided based upon precedent [9]. Fourth, few studies focus on change(s) in HRQoL impacts over time, which is particularly important to understand in progressive, life-limiting conditions. Finally, most studies investigating HRQoL (as a holistic construct) in this population are quantitative rather than qualitative, favouring quantification over in-depth insight (which can inform appropriate content validity of theoretical/measurement models). Qualitative and/or mixed-methods evidence is underrepresented and could provide more enriching and comprehensive information about the HRQoL of informal carers in rare, progressive, paediatric, life-limiting conditions. Such research is recognised as being potentially useful for patient advocacy in HTA submissions and/or as the building block of future measurement work, if such work is required.

### Challenge #3: methods of modelling carer HRQoL in cost-effectiveness analyses for HTA are underdeveloped and can lead to counterintuitive results

A third challenge are the issues involved in modelling carer HRQoL in cost-effectiveness analyses for HTA [9]. Modelling carer HRQoL and utility in cost-effectiveness models generates several difficulties. The methodological challenges have been outlined in detail elsewhere [9, 21], but ones that particularly apply for this group of carers include: (i) modelling bereavement; (ii) deciding on number of carers; and (iii) modelling carers’ HRQoL trajectories over time. First, in economic models, the choice of how to estimate informal carers' HRQoL following patient death is controversial and ethically problematic. Excluding carers’ HRQoL following patient death potentially devalues the bereaved carer. Alternatively, in the absence of good quality data, assumptions are often made about carer HRQoL following patient death, which can lead to counterintuitive results. For example, assuming carer HRQoL improves following patient death (due to the removal of the negative impacts of caring on HRQoL) makes interventions that prolong patient life seem less cost-effective (and therefore less desirable) [21]. While this and other issues affect all carers, this is particularly problematic in areas with higher carer burden and earlier mortality, such as rare, progressive, paediatric, life-limiting conditions. Second, deciding on the number of carers’ whose HRQoL is affected and thus should be included in a model is difficult. Often there is no good quality data to inform this, and, for HTA, considerations need to be made about ensuring comparability and consistency in processes adopted across different health conditions [22]. Third, understanding and modelling how carers’ HRQoL changes over time (particularly in progressive, life-limiting conditions) is difficult in the absence of good quality data, which is typically the case [9].

This challenge (methodological issues of modelling carer HRQoL in HTA) is an inhibiting factor for the inclusion of informal carer HRQoL data in HTA. Concerns have been raised over the value in capturing carer HRQoL for HTA if it cannot be modelled appropriately and is not received well by regulators. For example, where is the value for industry in collecting carer HRQoL data in trials if it counterintuitively makes a life-extending health technology look less cost-effective than otherwise by capturing a disutility associated with extended caregiving burden? Alongside potential solutions for research programmes to address some of these modelling issues [9], working with carers to understand their views on the use and potential consequences of including carer HRQoL in economic models could be beneficial.

## Future research priorities

**Fig. 1:**
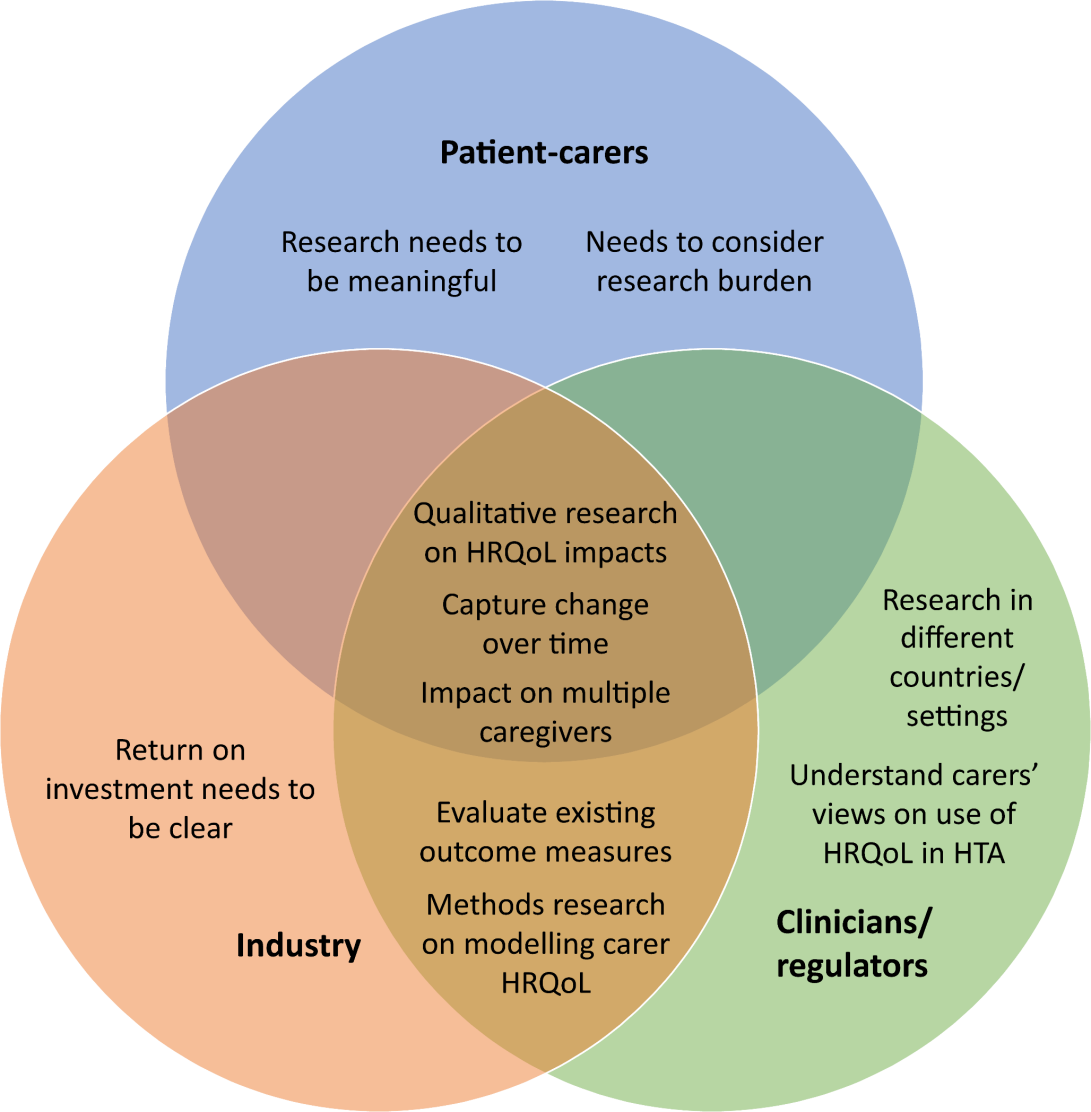
Examples of research priorities that may be relevant to different stakeholders for evidence generation for informal carer HRQoL in rare, progressive, paediatric, life-limiting conditions

We argue that a future research agenda for understanding and assessing HRQoL in informal carers of children and adolescents living with rare, progressive, life-limiting conditions in the context of HTA should focus first and foremost on addressing the three key challenges identified above. It is important to recognise that research priorities may differ for different stakeholder groups and Fig. 1 illustrates some of the potential research priorities that may be relevant to different stakeholders in the area. Targeted qualitative and/or mixed-methods studies should be conducted to address Challenge #2 and expand the underlying evidence base in this area. This includes studies that focus on commonalities across informal carers of children and adolescents living with different rare, progressive, life-limiting health conditions that can help inform a theoretical model of HRQoL that characterises impacts for this group of carers and is distinct from a wholly generic approach. This has been termed ‘disease-group-specific’, by focusing the measurement of HRQoL on a specific group of conditions or ones with similar impacts [11] (Challenge #1). For HTA, and a focus on comparability, there is benefit to having a HRQoL model that is applicable to multiple health conditions that share similar features, but not so generic that the content validity of the HRQoL model is inadequate.

Novel qualitative research should seek to clearly and definitively identify what is ‘different’ or ‘unique’ about being a carer of someone with a rare, progressive, paediatric, life-limiting condition, in terms of important HRQoL impacts, compared to a generic carer model (e.g., caring for older relatives with common health conditions). A particular consideration will be evidencing and understanding change of HRQoL impacts over time (e.g., after diagnosis, during transition stages and disease progression), which is important to understand in the context of an economic model built for HTA submissions. A second key consideration is the potential impacts for different informal carers (beyond the primary carer), including identifying the number of carers that are typically affected (required for cost-effectiveness modelling) and how they are affected. Identifying the relative HRQoL impacts for wider members of the family is an important consideration that has hitherto been overlooked in qualitative HRQoL research in this area. As well as parents, this may include siblings, grandparents, and others who take part in informal caregiving and/or whose HRQoL is affected significantly by the health condition.

Following the generation of a comprehensive model of HRQoL in informal carers of children and adolescents living with rare, progressive, life-limiting conditions through qualitative research, reviews of existing outcome measures could be conducted to establish their adequacy for use in this population. Comparisons can be made to the model unearthed from qualitative data, as well as assessments of the available evidence of their measurement properties for use in this population, for example, using the COnsensus-based Standards for the selection of health Measurement Instruments (COSMIN) procedures [23]. The goal of this research would be to identify if there is need to develop or adapt a specialised outcome measure for capturing HRQoL in informal carers of children and adolescents with rare, progressive, life-limiting conditions (i.e., taking a ‘disease-group-specific’ approach [11]) or whether existing instruments are satisfactory.

Finally, methodological research is needed to resolve known issues of modelling carer HRQoL in cost-effectiveness analyses and how to deal with potential problems that arise (e.g., counterintuitive results and how best to model utilities for bereavement) (Challenge #3). This could include group-based consultation exercises (i.e., with experts and decision-makers) and an appeal to expert advice (e.g., from the NICE Decision Support Unit) on how to best address methodological difficulties in modelling carer HRQoL. The ideal output would be a ‘best practice’ framework for people preparing HTA submissions to work from when modelling carer HRQoL. In all of these research endeavours, informal carers should be actively involved and informed, wherever possible. For example, through involvement as collaborators and advisors on projects.

Of course, in pursuing this research agenda key methodological decisions will need to be made. For example, deciding which conditions are included for study under the umbrella of ‘rare, progressive, paediatric, life-limiting’ is not necessarily straightforward. It would be challenging to include *all* possible conditions, so pragmatism and a focus on more prevalent conditions may be needed. The context and setting of research are important, with known differences between countries’ health and care support systems. Moreover, consideration of ethical issues and burden should be central to future research activities. For example, while it is important to capture caregiving impacts at the beginning, middle, and near-the-end of caring, it may be considered inappropriate to do so at sensitive times, such as at the time of diagnosis or bereavement and, instead, retrospective methods may need to be applied. Support for informal carers involved in research should be provided, such as access to counsellors if needed. Ultimately, there is an altruistic element to participation in research for informal carers and clearly identifying and articulating the way that the research will help the rare disease community is essential.

## Conclusion

Our understanding and use of informal carer HRQoL data in HTA submissions in rare, progressive, paediatric, life-limiting conditions is underdeveloped. Use of this data in HTA is hampered by key challenges, including: (i) the lack of applicability and/or content validity of a generic model of HRQoL for this group; (ii) limited evidence addressing specific areas of HRQoL impact in these informal carers, including commonalities across conditions, impact beyond the primary carer, and differences over time; and (iii) methodological challenges in modelling informal carer HRQoL in cost-effectiveness analyses. A research agenda is presented that addresses these challenges by focusing first on in-depth qualitative research to develop an appropriate, content valid ‘disease-group-specific’ model for understanding informal carer HRQoL in rare, progressive, paediatric, life-limiting conditions. This model can be used to inform the appropriate measurement of HRQoL in this population, which, alongside methodological research on addressing modelling challenges, can help to facilitate the recommended inclusion of informal carer HRQoL data in HTA submissions for children and adolescents living with rare, progressive, life-limiting conditions.
